# Potential user interest in new long-acting contraceptives: Results from a mixed methods study in Burkina Faso and Uganda

**DOI:** 10.1371/journal.pone.0217333

**Published:** 2019-05-28

**Authors:** Rebecca L. Callahan, Aurélie Brunie, Amelia C. L. Mackenzie, Madeleine Wayack-Pambè, Georges Guiella, Simon P. S. Kibira, Fredrick Makumbi

**Affiliations:** 1 Contraceptive Technology Innovation, Durham, NC, United States of America; 2 Health Services Research, Washington DC, United States of America; 3 Institut Supérieur des Sciences de la Population, Université de Ouagadougou, Ouagadougou, Burkina Faso; 4 School of Public Health, College of Health Sciences, Makerere University, Kampala, Uganda; Centers for Disease Control and Prevention, UNITED STATES

## Abstract

Method-related concerns represent an important cause of contraceptive non-use and discontinuation. User preferences must be incorporated into the design of new contraceptive technologies to ensure product success and improve family planning outcomes. We assessed preferences among potential users in Burkina Faso and Uganda for six contraceptive methods currently under development or ready for introduction: a new copper intra-uterine device (IUD), a levonorgestrel intra-uterine system, a new single-rod implant, a biodegradable implant, a longer-acting injectable, and a method of non-surgical permanent contraception. Questions were added to nationally-representative PMA2020 household surveys that asked 2,743 and 2,403 women in Burkina Faso and Uganda, respectively, their interest in using each new method. We assessed factors associated with interest through multivariable logistic regression models. We conducted qualitative interviews and focus groups with 398 women, 78 men, and 52 family planning providers and key informants to explore perceived advantages and disadvantages of the methods. Respondents expressed interest in using all new methods, with greatest interest in the longer-acting injectable (77% in Burkina Faso, 61% in Uganda), followed by a new single-rod implant. Least interest was expressed in a new copper IUD (26% Burkina Faso, 15% in Uganda). In both countries, women with less education had higher odds of interest in a longer-acting injectable. Interest in most new methods was associated with desiring a method lasting longer than one year and acceptance of lack of menstrual bleeding as a contraceptive side effect. Perceived advantages and disadvantages were similar between countries, including concerns about menstrual side effects and fear of the biodegradable nature of the biodegradable implant. Potential users, their partners, and providers are interested in new longer-acting methods, however, familiar forms including the injectable and implant may be the most immediately acceptable. A biodegradable implant will require clear counseling messages to allay potential fears.

## Introduction

Nearly 60 years have passed since the United States Food and Drug Administration (US FDA) approved the first hormonal contraceptive, the oral pill, Enovid. The importance of this landmark advancement in pharmaceutical science for the health and wellbeing of women and communities cannot be oversold. Contraception has been rightly heralded as one of the most important public health achievements in history, having positive effects on nearly every facet of society [1–3]. In the decades immediately following approval of the “Pill”, numerous other contraceptive agents and delivery devices were developed including new oral contraceptive formulations, injectable methods, implants, and intra-uterine devices (IUDs). This expanded method mix has been crucial for increasing contraceptive prevalence [4] and reaching users all over the world who have different contraceptive needs and preferences.

The pharmaceutical industry has reduced contraceptive research and development (R&D) efforts due to perceived low return on investment and relatively high regulatory hurdles [5]. Industry efforts over the last two decades have been limited to new oral pill combinations and iterations of the hormone-releasing IUD. The most recent new contraceptive approved by the US FDA in August of 2018, the year-long vaginal ring Annovera, was developed almost exclusively with public and foundation funding by the non-profit organization, Population Council [6].

However, more than 200 million women around the world still have an unmet need for contraception, i.e., they do not want to become pregnant but are not using contraception, and many are not using because of method-related reasons [7,8]. Misperceptions about pregnancy risk with infrequent sex or lack of a regular sexual partner sometimes lead to inconsistent or incorrect use [9]. Hormonal contraceptive methods such as oral pills and injectable methods are challenged by low adherence and/or low continuation rates in part due to need for frequent re-supply. In addition, too few acceptable long-acting methods are currently available to women who wish to limit or postpone childbearing for an extended time. With the number of women of reproductive age and desire to limit fertility increasing in many less developed regions [10–12], the need for new contraceptive technology is only growing.

The promise of new contraceptive technologies to meet this growing need has been bolstered recently with renewed interest from the philanthropic field, most notably the Bill & Melinda Gates Foundation, which has included investment in new contraceptive technologies as an area of focus within its family planning strategy [13]. Philanthropic and public sector donors, including the US Agency for International Development (USAID) and the US National Institutes of Health (NIH), made up nearly half of the $63 million invested in contraceptive R&D in 2013, the most recent year for which data are available [14]. One recipient of the Gates Foundation’s contraceptive grant funding is the human development organization, FHI 360. Through its Contraceptive Technology Innovation (CTI) Initiative launched in 2013, FHI 360 develops, evaluates, and introduces new and strategically important contraceptives with an emphasis on longer-acting methods [15]. The goal of the CTI initiative is to provide quality, affordable, and acceptable contraceptive products for those most at need in low-resource settings. Products in the CTI Initiative pipeline span multiple stages of research and development, targeting key opportunities for rapid expansion of the contraceptive method mix.

This research aimed to assess women’s, men’s, and family planning program staff’s attitudes toward specific contraceptive method attributes, their reactions to six new methods in the CTI Initiative pipeline, and drivers of method preferences including the decision-making context in two countries chosen to be broadly representative of Sub-Saharan Africa. The six methods included in this research are a new copper IUD (Cu-IUD), a levonorgestrel-releasing intra-uterine system (LNG-IUS), a new single-rod implant (SRI), a biodegradable implant (BDI), a longer-acting injectable (LAI), and a method of non-surgical permanent contraception (NSPC) (Table 1). Here we focus on preferences about these methods under development. Elsewhere we describe preferred product characteristics not specific to these methods to inform product development more broadly [16]. Specific objectives addressed in the present paper are to 1) estimate the level of potential interest in using the six methods, 2) explore factors affecting interest, and 3) identify method advantages and disadvantages from the perspective of potential female users, men, providers, and decision-makers.

**Table 1:** New contraceptive technologies included in the study.

Method		Summary Description*
New copper intrauterine device	Cu-IUD	A non-hormonal IUD with different shape and/or size and a use duration of 10 years
Levonorgestrel intrauterine system	LNG-IUS	A more-affordable alternative to LNG-IUS products currently on the market with a use duration of 5 years
New single rod implant	SRI	An alternative progestin-only implant with a use duration of 5 years
Biodegradable implant	BDI	A progestin-containing biodegradable implant with a use duration of 18 months and 12 months of removability
Longer-acting injectable	LAI	A hormonal injectable with a duration of 6 months
Non-surgical permanent contraception	NSPC	Alternative to surgical sterilization involving vaginal delivery of a tubal occluding agent

*Full descriptions read to respondents are shown in S1 Appendix

## Methods

**Fig 1:**
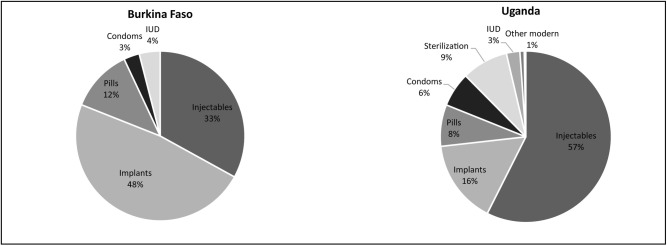
Modern contraceptive methods used by women in union aged 15–49 in Burkina Faso and Uganda [17,18].

We implemented a mixed methods study, including a quantitative survey and qualitative focus group discussions and in-depth interviews in Burkina Faso and Uganda. The two countries were chosen for this study because of their geographic locations, West and East Africa, respectively, and their participation in the Performance Monitoring and Accountability 2020 (PMA2020) survey program, described in more detail below. Burkina Faso and Uganda represent different cultural and socioeconomic contexts and contraceptive use patterns. While both have relatively low modern contraceptive prevalence rates (mCPRs) and high unmet need for contraception, their contraceptive methods mixes and family planning service delivery environments are different (Table 2 and Fig 1). More than 85% of women in Burkina Faso, across wealth quintiles, receive their contraceptive method from the public sector whereas in Uganda, a little more than half use the public sector [17,18]. In Uganda, community health workers (CHWs), called Village Health Teams (VHTs), offer condoms, pills, and injectables. Two-thirds of public sector service delivery points in Uganda support VHTs. In contrast, in Burkina Faso, CHWs, known as Agent de santé à base communautaire (ASBC), only distribute condoms and resupply oral contraceptives and only one-third of public sector service delivery points support ASBCs.

**Table 2:** Select family planning indicators in Burkina Faso and Uganda [17,18].

	Burkina Faso	Uganda
Total fertility rate (TFR)	6.0	6.0
Modern contraceptive prevalence rate (mCPR) among women in union aged 15–49	25%	32%
Unmet need for contraception among married women aged 15–49	29%	31%
Service delivery points (public and private) offering family planning	96%	95%
Public facilities offering any LARC*	94%	61%
Private facilities offering any LARC	43%	18%
Service delivery points (public and private) supporting community health workers (CHWs)	22%	41%

*Long-acting reversible contraception (i.e., implants and IUDs)

The study received ethical approval from the FHI 360 Protection of Human Subjects Committee in the US (768990), the Comité d'Ethique pour la Recherche en Santé in Burkina Faso (2016-10-120), and the Makerere University’s School of Public Health Higher Degrees, Research and Ethics Committee (00011373) and the Uganda National Council for Science and Technology in Uganda (SS4002). Written informed consent, or assent if 15–17 years old, was given by all participants. Per IRB requirements, parental consent was also required for non-emancipated minors in Uganda, but not required for emancipated minors, which includes married minors.

### Quantitative methods

The Performance Monitoring and Accountability 2020 (PMA2020) survey program implements repeated cross-sectional surveys in 11 countries to monitor key health and development indicators [19]. PMA2020 uses female resident enumerators (REs) and a mobile-assisted data collection system to collect nationally representative data at the household and facility level based on a multistage random sampling design. The household survey includes a household and women’s questionnaire. Eligibility criteria for the latter is any woman between the ages of 15–49 who is either a usual member of the household or who slept in the household the night before. Additional details about the PMA2020 survey methodology and sample size justification are available elsewhere [20,21].

We partnered with PMA2020 to add a set of 12 questions to the women’s questionnaire in Round 4 of the PMA2020 surveys in Uganda (April-May 2016) and Burkina Faso (November 2016-January 2017). Women who were not using a permanent method of contraception and who said they were interested in using any new contraceptive method if it became available in the future were asked the module of questions. In Burkina Faso, women who stated they thought they would use any contraceptive method in the future were also asked the module questions. As part of these questions, each of the six methods was described, and an image illustrating the method was shown to respondents after which, they were asked whether they would be interested in using the new method at some point in the future. Method descriptions and images included in the survey are available in S1 Appendix. Of the methods they expressed interest in using, respondents were then asked which they would most prefer to use. For current or recent users, i.e., those reporting having used a modern or traditional method in the last 12 months, the choice of their most preferred method also included their current/recent method.

The main outcome measure is the proportion of women who expressed interest in using each of the six methods. We examined interest in each method for all women and separately for women currently using contraception (current users), women who used contraception in the past but weren’t currently (past users), and women who have never used contraception (never users). Design-adjusted Rao-Scott tests were used to test for differences in interest to use the methods between user categories.

Choice of preferred method was examined separately for current/recent users and for other women. Women who did not express interest in any of the new methods were counted as preferring their current/recent method or no method, respectively. Women who only expressed interest in one of the new methods and were not current/recent users were attributed this method as their preferred one.

To report on factors affecting interest in the new methods, we conducted exploratory multivariable analyses using one logistic regression model per method. The dependent variable was interest in using the method (definitely or probably interested vs. definitely not or probably not interested). Covariates were selected from variables in the broader PMA2020 women’s questionnaire and two other questions from the acceptability module: preferred method duration (dichotomized, preferring more or less than/equal to one year of effectiveness) and acceptability of amenorrhea as a contraceptive side effect (i.e., “With some contraceptive methods, women do not get their period, but their period and their fertility return when they stop using it. Would you choose a method that stops your period?”). Selection of covariates into the models was informed by theoretical considerations and descriptive and bivariate analyses. All analyses were conducted using Stata 14 and adjusted for the complex sampling design and unit nonresponse to the women’s questionnaire using sampling variables and weights provided with the dataset. Methods appropriate for subpopulation analysis were used. We considered a p-value of 0.05 or below to be statistically significant.

### Qualitative methods

We conducted focus group discussions (FGDs) with women aged 15–49 (married adolescents aged 15–17 and married and unmarried women aged 18–49) and men aged 18 and older in four of the 13 regions of Burkina Faso (North, East, Boucle du Mouhoun and South-West) plus Ouagadougou and each of the four regions of Uganda (Eastern, Western, Northern, and Central) plus Kampala City. We also conducted in-depth interviews (IDIs) with family planning providers (from the public sector only in Burkina Faso and from the public and private sectors in Uganda) in the same areas, and with family planning program staff and government officials (key informants) in some of the regions and at the central level. In each region, a PMA2020 sampling area was selected based on logistical and practical considerations to conduct the FGDs and IDIs. In Burkina Faso, providers at local health centers mobilized FGD participants, whereas in Uganda we relied on Village Health Teams and some men were also recruited through their female partners who took part in FGDs or through a snowball approach.

In Uganda, seven FGDs (two with women using long-acting reversible contraception (LARC) methods (i.e., implants and IUDs), two with women using other modern methods, two with women not using a method, and one with men) and five IDIs with providers were conducted in each sampling area. In Burkina Faso, FGDs were not divided by type of modern method used so five were conducted in each area (two with modern method users, two with non-users, and one with men), as well as three IDIs with providers. There were seven key informant interviews in Burkina Faso, and eight in Uganda. All interviews were conducted by trained interviewers who were native speakers of the predominant local language of the selected sampling area using pre-tested topic guides. In Burkina Faso, FGDs were conducted in Gulmancéma, Dagara, Dioula, San, or Mooré and in Uganda languages included Luganda, Lugbara, Lusoga, or Runyankore. IDIs with providers and key informants were conducted in French in Burkina Faso and English in Uganda.

During the FGDs and IDIs, participants were presented with descriptions and images of the six products in the CTI Initiative pipeline (descriptions from the topic guides and images are shown in S2 Appendix); research assistants asked respondents how they felt about the method and then about what they liked and didn’t like about the method. For women, they also inquired about interest in using the method. Women and men participating in FGDs were offered a refreshment and received 20,000 shillings (USD 5.40) in Uganda and soap in Burkina Faso. Providers received 12,000 shillings (USD 3.25) in Uganda and a refreshment in Burkina Faso. Key informants were not compensated.

All FGDs and IDIs were audio-recorded and the recordings transcribed into French in Burkina Faso and English in Uganda. Transcripts were coded using NVivo 11 and analyzed thematically. We wrote detailed memos describing sub-themes related to each main code, including perceived advantages and disadvantages of each of the six new methods. We also created matrices in Excel to examine variations in sub-themes by country and participant type.

## Results

### PMA2020 survey results

In Burkina Faso, 2,743 of the 3,203 women (86%) who completed the PMA2020 also completed the contraceptive acceptability module of questions. In Uganda, 2,403 of the 3,793 PMA2020 respondents (63%) completed the acceptability module. The response rates among eligible women were 99.8% and 100% in Burkina Faso and Uganda, respectively.

Respondent characteristics are shown in Table 3. Mean age (~27 years) and parity (~3) and proportion married (~75%) were similar in the two countries, however, more than 60% of women in Burkina Faso reported having no schooling compared with less than 10% in Uganda. One third of women in Uganda and one quarter of respondents in Burkina Faso reported use of a modern method of contraception at the time of the survey. A little under one fifth of women in Burkina Faso and about a quarter of women in Uganda were not current users but had used contraception in the past. Nearly three-fifths of respondents in Burkina Faso had never used contraception compared with two fifths in Uganda.

**Table 3:** Characteristics of PMA2020 survey respondents who completed the acceptability module.

Characteristic (%)	Burkina Faso (n = 2,743)	Uganda (n = 2,403)
**Age**		
15–24	40.4	44.6
25–34	33.8	36.1
35–49	25.8	19.4
mean age (SE)	27.9 (0.3)	27.0 (0.2)
**Residence**		
Urban	23.8	18.2
Rural	76.2	81.8
**Highest education attended**		
None	62.5	6.0
Primary	17.2	64.0
Secondary	18.0	24.6
More than secondary	2.4	5.4
**Marital status**		
Never married	20.3	17.9
Married/cohabitating	75.9	72.6
Divorced/separated/widowed	3.7	9.5
**Parity**		
0	22.3	18.7
1–2	26.1	31.2
3–4	22.6	22.3
5+	29.0	27.8
mean number of children (SE)	3.1 (0.1)	3.1 (0.1)
**Contraceptive use**		
Never user	56.0	37.9
Past user	17.6	25.5
Current modern short-acting user	13.0	27.1
Current modern long-acting user	12.3	6.0
Current traditional user	1.1	3.5
**Prefer method lasting >1 year**	61.3	60.6
**Finds contraceptive-induced amenorrhea acceptable**	65.0	39.9

**Fig 2:**
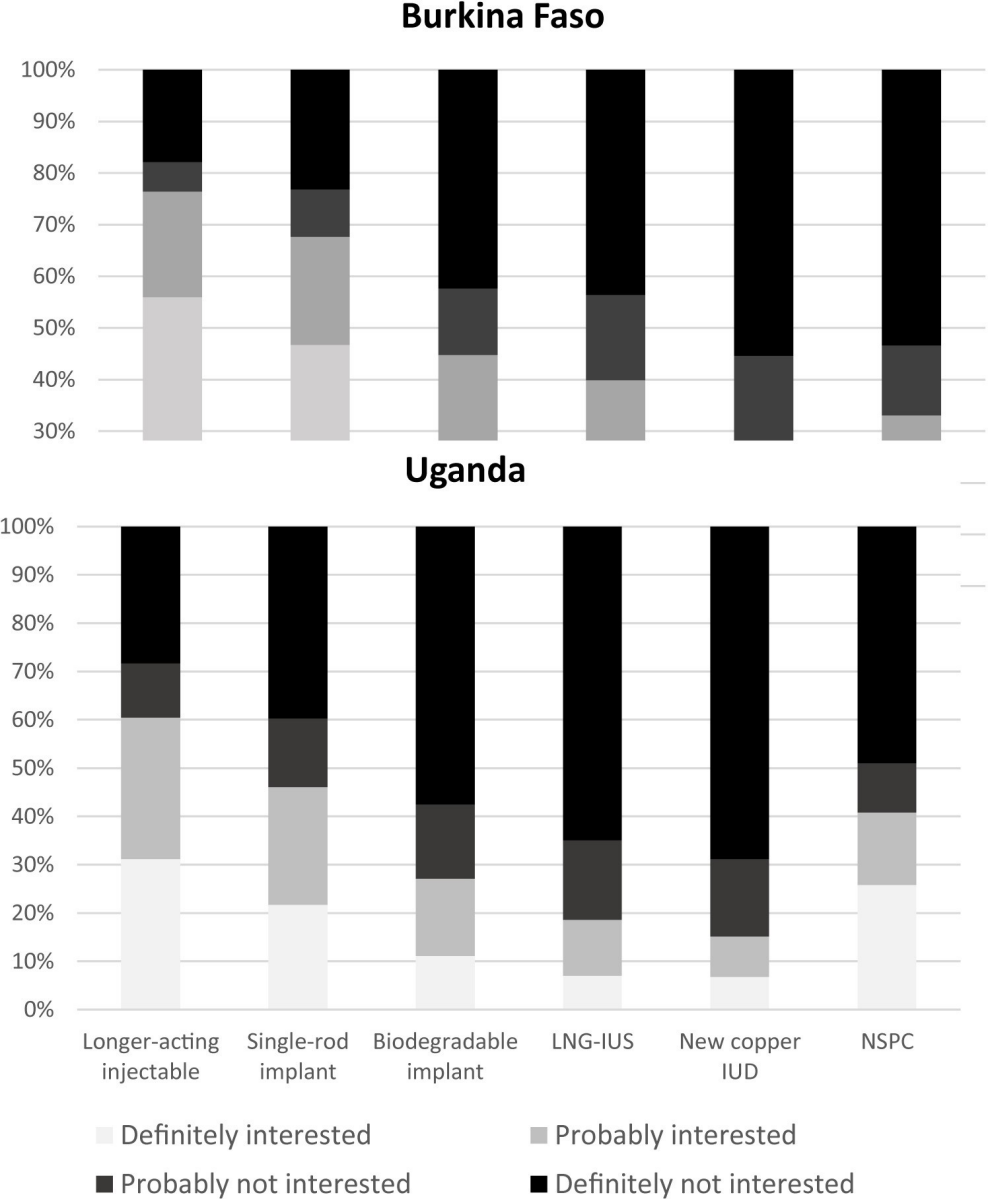
Reported interest in using the six new methods at some point in the future if they were available.

Fig 2 shows that 77% of women in Burkina Faso and more than 60% of women in Uganda indicated that they would “definitely” or “probably” be interested in using the LAI. Close to 70% of women in Burkina Faso and just under half of women in Uganda indicated they would use a new SRI. Conversely, 60 and 74% of respondents in Burkina Faso and 81 and 85% in Uganda said they would not use the LNG-IUS and new Cu-IUD, respectively. Except for NSPC, a larger proportion of women in Burkina Faso expressed interest in using the new methods compared with Uganda. When comparing never, past, and current users of contraception, the difference in the proportion stating they would use one of the new methods was statistically significant only for NSPC in Uganda, for which 36, 46, and 42% of never, past, and current users, respectively said they would use the method (results not shown).

**Fig 3:**
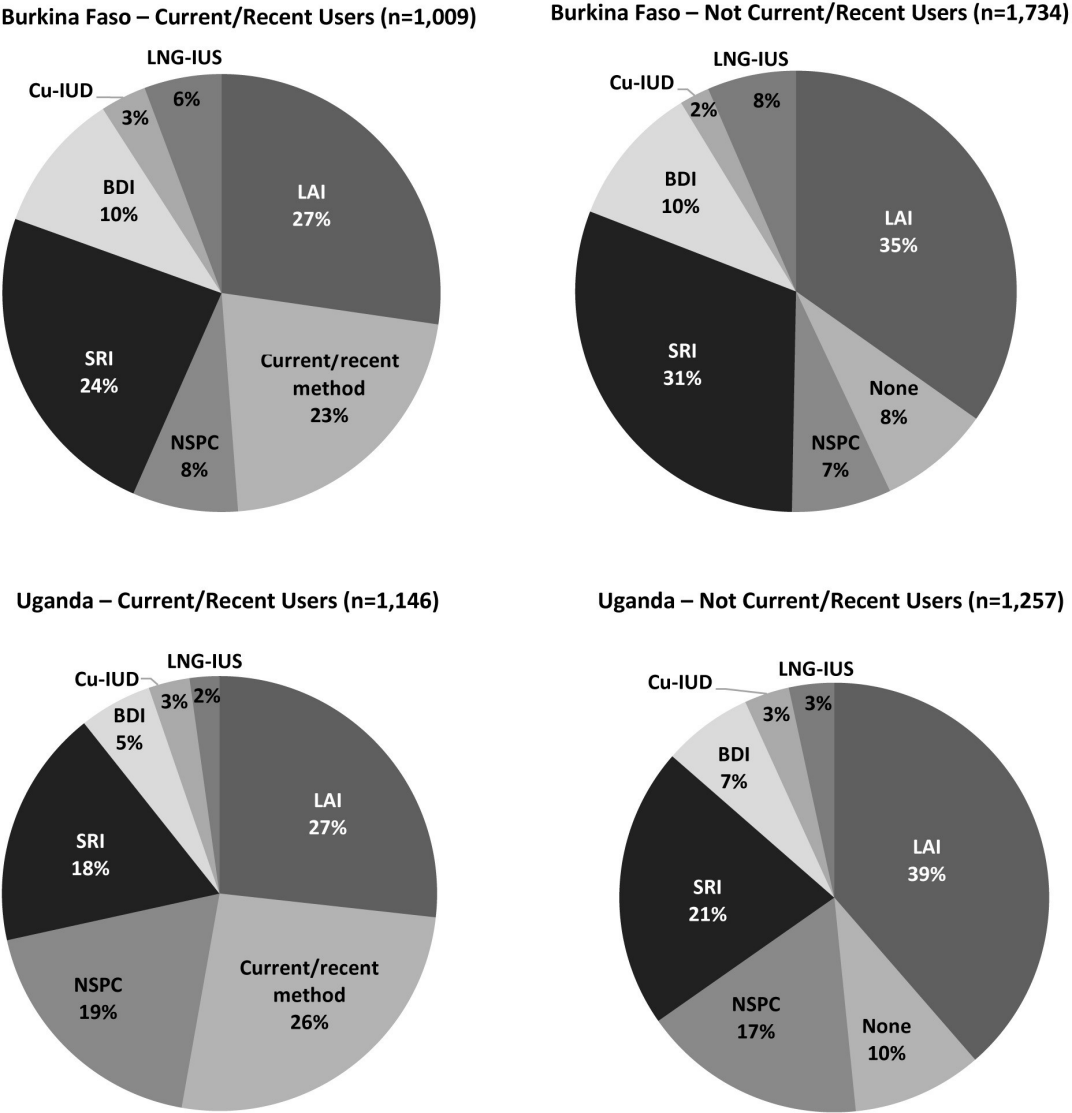
Most preferred method by country and current contraceptive use status.

Fig 3 shows the methods current/recent and none/past users said they would most be interested in using. Among the former group in both countries, nearly 75% would choose one of the new methods while only one quarter said they would continue with their current or recent method. Among non-users in both countries, 90% would choose one of the new methods and only 10% would choose none of the methods. The LAI was the most preferred method among both groups in both countries. In Burkina Faso, the second most preferred method among both user groups was the SRI. In Uganda, approximately 20% of all women preferred NSPC and a similar proportion preferred the SRI.

Odds ratios (ORs) from multivariable analyses describing associations between several demographic characteristics and method characteristic preference variables (i.e., acceptability of contraceptive-induced amenorrhea and preference for a method lasting longer than one year) and interest in using each of the new methods are shown in Tables 4 and 5. Women in both countries who found contraceptive-induced amenorrhea to be acceptable had higher odds of interest in each method except for NSPC in Burkina Faso (see Tables 4 and 5 for ORs and 95% confidence intervals [95% CIs]). Also, in both countries, women who preferred a method lasting longer than one year had lower odds of interest in the LAI and higher odds of interest in the other methods except for the BDI and Cu-IUD in Burkina Faso (see Tables 4 and 5 for ORs and 95% CIs). Compared to women who attended primary or no school, women who attended secondary school or higher had lower odds of interest in using the LAI in both Uganda (OR = 0.8, 95% CI = 0.6–1.0) and Burkina Faso (0.7, 0.5–1.0), as did women in the wealthiest quintile in Uganda (0.6, 0.3–0.9, compared to women in the lower quintile). Women had higher odds of interest in this method and the LNG-IUS if they were living in rural areas of Burkina Faso (1.5, 1.0–2.3). In Uganda, women over 24 and women living in rural areas had higher odds of being interested in NSPC (see Tables 4 and 5 for ORs and 95% CIs). Women who wanted more children tended to have lower odds of interest in NSPC in both countries (see Tables 4 and 5 for ORs and 95% CIs).

**Table 4:** Adjusted odds ratios for expressing interest in using the new methods among women in Burkina Faso (n = 2,618).

Characteristic (reference group)	LAI	SRI	BDI	LNG-IUS	Cu-IUD	NSPC
	OR (95% CI)					
Age (15–24)						
25–34	0.8 (0.6–1.2)	0.9 (0.6–1.4)	1.4 (1.0–1.9)	0.9 (0.7–1.2)	1.1 (0.8–1.6)	0.8 (0.6–1.2)
35–49	**0.6 (0.4–0.9)**	0.6 (0.4–1.1)	0.9 (0.5–1.3)	0.6 (0.4–1.0)	1.1 (0.6–1.9)	0.8 (0.5–1.3)
Residence (Urban)						
Rural	**1.5 (1.0–2.3)**	1.1 (0.7–1.9)	1.0 (0.6–1.7)	**1.6 (1.0–2.4)**	1.5 (0.9–2.4)	1.2 (0.8–2.0)
Education (≤primary)						
≥Secondary	**0.7 (0.5–1.0)**	**0.7 (0.5–0.9)**	0.7 (0.5–1.1)	0.9 (0.6–1.2)	0.8 (0.6–1.0)	0.89 (0.6–1.3)
Wealth (lowest)						
Middle	1.1 (0.7–1.8)	0.8 (0.6–1.1)	1.1 (0.8–1.5)	0.8 (0.6–1.2)	0.8 (0.6–1.1)	0.9 (0.6–1.3)
Highest	0.9 (0.6–1.3)	0.7 (0.4–1.1)	0.8 (0.5–1.3)	1.1 (0.7–1.7)	1.1 (0.7–1.7)	0.9 (0.5–1.4)
Marriage (never married)						
Married	1.1 (0.7–1.9)	1.2 (0.8–1.8)	1.6 (1.0–2.5)	1.2 (0.7–1.8)	1.0 (0.6–1.8)	1.0(0.6–1.7)
Div./Sep./Wid.	1.0 (0.5–2.3)	0.7 (0.4–1.5)	1.6 (0.7–3.5)	1.6 (0.9–3.0)	1.0 (0.5–2.0)	1.0 (0.5–2.0)
Parity (0)						
1–2	1.1 (0.6–1.9)	1.1 (0.7–1.6)	0.7 (0.4–1.1)	0.8 (0.5–1.3)	1.0 (0.6–1.7)	1.1 (0.6–2.0)
3–4	1.0 (0.6–1.8)	1.5 (0.9–2.5)	0.7 (0.4–1.3)	1.1 (0.7–1.7)	1.2 (0.7–2.1)	1.6 (0.8–3.2)
5+	1.1 (0.6–2.0)	1.3 (0.7–2.5)	0.8 (0.4–1.4)	1.1 (0.6–1.9)	1.2 (0.6–2.3)	1.8 (0.9–3.7)
Fertility intentions (no more)						
Child in < 2 years/undecided	1.3 (0.9–2.0)	1.0 (0.7–1.5)	**1.5 (1.1–2.0)**	1.1 (0.8–1.7)	0.8 (0.5–1.1)	**0.5 (0.3–0.8)**
Child in 2+ years	**1.5 (1.0–2.1)**	1.1 (0.8–1.6)	1.2 (0.8–1.7)	0.9 (0.6–1.3)	**0.6 (0.5–0.9)**	**0.4 (0.3–0.6)**
Contraceptive use* (never user)						
Past user	1.0 (0.7–1.4)	0.8 (0.6–1.0)	0.8 (0.6–1.2)	0.8 (0.6–1.1)	0.8 (0.5–1.3)	1.2 (0.8–1.7)
Current user	1.1 (0.8–1.5)	1.1 (0.9–1.4)	1.1 (0.8–1.5)	1.1 (0.8–1.5)	1.1 (0.8–1.6)	1.3 (0.9–1.6)
Prefer long-acting duration**	**0.7 (0.5–0.9)**	**2.6 (2.0–3.5)**	1.1 (0.8–1.4)	**1.5 (1.1–1.9)**	1.1 (0.9–1.4)	**1.4 (1.0–2.0)**
Amenorrhea acceptable	**2.8 (2.1–3.7)**	**2.1(1.6–2.7)**	**2.0 (1.4–2.7)**	**2.1 (1.6–2.9)**	**2.2 (1.6–3.2)**	1.4 (1.0–2.0)

*of any contraceptive method, modern or traditional

**1 year or longer, including permanent; Statistically significant values (p ≤ 0.05) are bolded.

**Table 5:** Adjusted odds ratios for expressing interest in using the new methods among women in Uganda (n = 2,292).

Characteristic (reference group)	LAI	SRI	BDI	LNG-IUS	Cu-IUD	NSPC
	OR (95% CI)					
Age (15–24)						
25–34	0.9 (0.6–1.3)	1.1 (0.8–1.5)	0.9 (0.7–1.3)	1.1 (0.8–1.7)	1.1 (0.7–1.7)	**1.4 (1.0–1.9)**
35–49	0.7 (0.4–1.1)	0.9 (0.6–1.4)	1.0 (0.6–1.5)	1.0 (0.6–1.8)	0.9 (0.5–1.7)	**1.7 (1.1–2.7)**
Residence (Urban)						
Rural	0.8 (0.5–1.3)	1.1 (0.8–1.5)	1.5 (0.9–2.5)	1.3 (0.8–2.1)	1.0 (0.5–1.8)	**1.7 (1.2–2.4)**
Education (≤primary)						
≥Secondary	**0.8 (0.6–1.0)**	0.9 (0.7–1.2)	0.7 (0.5–1.0)	0.9 (0.7–1.2)	1.0 (0.7–1.4)	0.9 (0.6–1.2)
Wealth (lowest)						
Second	1.0 (0.7–1.4)	1.2 (0.8–1.7)	1.1 (0.7–1.7)	1.0 (0.7–1.5)	0.7 (0.5–1.1)	1.2 (0.7–1.9)
Middle	1.3 (0.9–2.0)	1.0 (0.7–1.6)	0.9 (0.6–1.3)	1.2 (0.7–2.0)	1.0 (0.5–1.8)	1.5 (0.9–2.4)
Fourth	1.1 (0.7–1.7)	1.1 (0.7–1.7)	1.1 (0.7–1.7)	1.4 (0.8–2.3)	0.8 (0.4–1.7)	1.2 (0.7–1.8)
Highest	**0.6 (0.3–0.9)**	0.8 (0.5–1.3)	1.0 (0.6–1.6)	1.1 (0.6–1.9)	1.0 (0.5–1.8)	1.6 (0.9–2.7)
Marriage (never married)						
Married	1.4 (1.0–2.0)	1.2 (0.8–1.9)	1.4 (0.9–2.1)	1 (0.6–1.5.0)	0.9 (0.5–1.7)	1.2 (0.7–2.0)
Div./Sep./Wid.	1.3 (0.8–2.3)	1.2 (0.7–2.1)	1.0 (0.6–1.6)	0.8 (0.4–1.5)	0.5 (0.2–1.1)	1.2 (0.7–2.1)
Parity (0)						
1–2	1.0 (0.7–1.5)	0.7 (0.5–1.1)	0.9 (0.6–1.4)	0.7 (0.4–1.2)	1.3 (0.6–2.9)	0.8 (0.5–1.4)
3–4	1.3 (0.8–2.1)	0.9 (0.5–1.5)	0.9 (0.6–1.6)	0.8 (0.5–1.5)	1.7 (0.7–4.0)	0.9 (0.6–1.6)
5+	0.8 (0.5–1.3)	0.6 (0.4–1.1)	0.7 (0.4–1.3)	0.5 (0.3–1.1)	1.0 (0.4–2.7)	1.3 (0.7–2.4)
Fertility intentions (no more)						
Child in < 2 years/undecided	1.1 (0.8–1.5)	0.9 (0.6–1.2)	1.2 (0.8–1.8)	0.8 (0.5–1.2)	0.7 (0.4–1.2)	**0.7 (0.5–0.9)**
Child in 2+ years	1.2 (0.9–1.6)	1.2 (0.9–1.6)	1.3 (0.9–1.8)	0.8 (0.6–1.1)	0.9 (0.6–1.4)	0.8 (0.6–1.1)
Contraceptive use* (never user)						
Past user	0.8 (0.6–1.1)	1.3 (1.0–1.7)	0.9 (0.7–1.3)	0.8 (0.6–1.2)	0.7 (0.4–1.0)	1.3 (0.9–1.9)
Current user	1.1 (0.8–1.5)	1.3 (1.0–1.7)	1.1 (0.8–1.6)	1.0 (0.7–1.5)	0.9 (0.6–1.3)	1.0 (0.7–1.5)
Prefer long-acting duration**	**0.6 (0.5–0.8)**	**3.1 (2.5–3.9)**	**1.7 (1.3–2.3)**	**1.7 (1.3–2.3)**	**1.4 (1.0–2.0)**	**1.6 (1.2–2.0)**
Amenorrhea acceptable	**2.7 (2.0–3.6)**	**1.5 (1.2–1.9)**	**1.6 (1.2–2.2)**	**1.5 (1.1–2.0)**	**1.7 (1.2–2.4)**	**1.4 (1.0–1.8)**

*of any contraceptive method, modern or traditional

**1 year or longer, including permanent; Statistically significant values (p ≤ 0.05) are bolded.

### Qualitative results

Characteristics of women and men participating in FGDs and family planning providers and key informants in the two study countries are shown in Tables 6 and 7, respectively. Mean age was similar across focus groups in both countries, while larger proportions of participants had no education in Burkina Faso. Both implant (60%) and IUD (40%) users participated in the FGD for LARC users in Uganda; 38% of participants in FGDs with users in Burkina Faso used an implant. Current nonuser FGDs included past and never users of contraception. Most providers interviewed in both countries worked in the public sector, had experience providing implants and IUDs, and had an average of nine years of in their current position.

**Table 6:** Characteristics of FGD respondents.

Characteristic	UGANDA				BURKINA FASO		
	LARC users (n = 68)	Other Users (n = 88)	Current Nonusers^a^ (n = 82)	Men (n = 38)	Users (n = 79)	Current Nonusers^a^ (n = 81)	Men (n = 40)
**Mean age**	32	31	31	38	30	28	39
**Mean parity**	4	4	4	5	4	3	5
**Marital status (%)**							
Single	0	5	6	0	0	2	2
Married	57	75	52	68	99	88	98
In union	35	20	37	32	1	6	0
Other	7	1	5	0	0	4	0
**Highest education attained (%)**							
None	16	8	27	50	78	77	50
Some primary	51	67	48	30	16	17	30
Some secondary	32	25	26	20	5	6	20
**Current modern method use (%)**							
None	0	0	100	18	0	100	33
Condoms	0	0	0	20	1	0	10
Pills	0	17	0	8	15	0	8
Injectable	0	82	0	48	46	0	15
Implant	60	0	0	8	38	0	20
IUD	40	0	0	0	0	0	3
Female sterilization	0	1	0	0	1	0	0
Unsure	0	0	0	0	0	0	13

^**a**^ Current nonusers includes past users and never users of contraception.

**Table 7:** Characteristics of IDI respondents.

	UGANDA		Burkina faso	
	Providers (n = 22)	Key informants (n = 7)	Providers (n = 15)	Key informants (n = 8)
Mean age	37	—	37	—
Mean years in current position	9	—	9	12
Sector, n				
Public	13	4	15	6
Private or NGO	9	3	0	2
Sex, n				
Male	4	5	5	5
Female	18	2	10	3
Provides implants, n				
Yes	18	—	14	—
No	4	—	1	—
Provides IUD, n				
Yes	16	—	10	—
No	6	—	5	—

Here we describe the perceived advantages and disadvantages of each of the six methods, in the order the methods were presented to participants. The most commonly cited method characteristics are shown in Table 8. Some characteristics were identified as both advantages and disadvantages.

**Table 8:** Advantages and disadvantages of the six new methods as described by women participating in FGDs in Uganda and Burkina Faso.

LAI	SRI	BDI	LNG-IUS	Cu-IUD	NSPC
*Advantages*					
• **6-month duration** • Injectable delivery • Discreetness • Ease of access	• 5-year duration • Potential early removal • Only one rod	• No removal	• **Treatment for menorrhagia** • **5-year duration**	• Potential early removal • No hormones	• Non-surgical • No effect on period
*Both Advantage and Disadvantage*					
		• 1.5-year duration	• Lighter periods/ amenorrhea	• 10-year duration	• Permanence
*Disadvantage*					
• Bleeding irregularities • Perceived side effects • Non-reversible for 6 months	• Perceived side effects •**Irregular bleeding**	• Irregular bleeding • **Dissolves**	• Hormones • Uterine location	• **Heavier periods** • Uterine location • Pain • Potential expulsion	

Bold indicates mention in more than half of focus groups

#### Advantages and disadvantages of the new copper IUD

Some focus group participants, as well as providers, liked the non-hormonal nature of the Cu IUD. Several FGD participants and providers linked the lack of hormones to the potential for fewer side effects, including in Uganda to an immediate return to fertility. Some providers in both Uganda and Burkina Faso mentioned that a smaller IUD may be easier to insert and cause less pain and bleeding, which may increase acceptability among users.

Opinions on the 10-year duration of the Cu IUD were mixed across participant groups, although more women in Uganda liked than disliked the extended timeframe. Diverging perspectives on whether or not ten years was perceived as good were linked to the perception that the method may be better suited to limit than to space births, and sometimes to contrasting views on removals. Many women and some men in both countries appreciated the option of an early removal. On the other hand, one provider in Burkina Faso said that women may fail to understand that the method can be removed early, and a few participants in both countries mentioned possible challenges with access to removal services.

*My fear is the expense involved in removing it. They may insert it free of charge but when time comes for us to have it removed, in [public hospital], they will charge us ten thousand shillings*. *That aside you will need transport which brings the amount to 50,000 shillings. Now in case you did not involve your husband where will one get it?*

- Injectable user with four children in Central region, Uganda

In almost all FGDs with women and men in Burkina Faso, approximately half of FGDs in Uganda, and several IDIs with providers, participants highlighted the potential for heavy bleeding with the copper IUD as a strong disadvantage, largely because of concerns over potential adverse health effects and/or interference with their daily lives. A 30-year old mother of three who had never used contraception in Burkina Faso indicated that lack of quality food in combination with increased menstrual bleeding would be detrimental to health.

*Here our food is not what you think, so if your blood is going to flow a lot, it is not going to be fine*.

A number of women in both countries and some men in Burkina Faso mentioned the uterine placement of the IUD as a disadvantage with the perception that it could interfere with sex and cause uterine pain or other problems, potentially jeopardizing fertility. In Uganda, a number of women and several providers also mentioned fear of expulsion of the device, which in some cases they thought may be facilitated by the heavy bleeding. In several discussions, particularly, in Uganda, women shared negative anecdotes of copper IUD use experience and described rumors they had heard about the method causing cancer and other harmful health effects.

#### Advantages and disadvantages of the LNG-IUS

The ability to treat heavy or painful menstruation was widely perceived as a benefit of the LNG-IUS across groups. Many users and providers and some men and key informants said they liked the long-acting, 5-year duration, particularly because they found it to be more reasonable in contrast to the 10 years of the new copper IUD.

The possibility that the method may lead to lighter periods was also viewed favorably by women, men, and providers, although participants had divergent opinions on amenorrhea. Some women feared harmful effects of not having a regular period. One key informant in Burkina Faso compared bleeding side effects to those from the existing copper IUD, saying,

*With the copper IUD*, *many women complain about heavy bleeding during menstruation*. *Menses last longer and the flow becomes more than normal*. *So*, *if we could have a hormonal IUD that can reduce the flow and reduce cramps*, *it will be welcome*, *many side effects will reduce and women will adhere to this IUD*.*–*Regional Health Director in Burkina Faso

Dislike of uterine placement was reiterated in several FGDs. While a few participants, primarily providers, liked that the method was hormonal on grounds of perceived effectiveness, in Uganda, many women, a few men, and some providers also associated hormones with side effects.

*It has a problem because it contains hormones so if you have failed to use the injectable you may fail to use it*.*–*LARC user in Southwestern Region, Uganda

#### Advantages and disadvantages of the new single rod implant

Providers in both countries and women and men, mostly in Uganda, talked about the five-year duration as an advantage of the method both in relation to spacing benefits and reduced clinic visits. In several FGDs with women in both countries and most FGDs with men in Uganda, participants also appreciated having the option of an early removal. Some women and many providers liked the idea of a single rod. Providers in both countries and most key informants in Burkina Faso noted that the insertion and removal procedure would be easier with a single rod compared with a two-rod system.

*What we have*, *it is Jadelle*, *the two rods*, *now if it gets back to one*, *it is good because to remove two*, *it’s whatchamacallit…it will be easier…in any case*, *the trauma…it won’t be the same thing*, *and it also reduces the time for inserting or removing…with five years*, *it is good*, *it is good*.*–*CSPS provider in Burkina Faso

Interviews highlight only one primary disadvantage of the single rod implant. Many women, men, and some providers viewed the potential for irregular bleeding as undesirable. Some users in Uganda were also concerned about having a foreign body inserted in their arm particularly because of fear that it could move in the body or cause arm pain that could interfere with their ability to work. A 49 year-old mother of five who was currently using an implant in West Nile Region said,

*Why I don’t like it is if you do heavy work like digging you feel pain around the spot where the method is inserted*.

#### Advantages and disadvantages of the biodegradable implant

Perspectives on several features of the biodegradable implant, including its duration and its biodegradable nature, were split. Opinions on the 1.5-year duration of the biodegradable implant were divided across participant groups between those who said it was good for spacing and those who considered the duration too short.

Many women, especially in Uganda, some men, and providers and key informants noted the fact that the implant would not have to be removed as an advantage. Without removal, benefits to women would include avoiding potential pain from an additional invasive procedure, not having to make an additional clinic visit, and saving money. Some providers and key informants also liked that it would save on resources for removal. In Uganda, several women said they liked that the implant could be removed during the first year, while a few providers were concerned about removal not being possible in the remaining six months.

Overall, however, respondents were generally more concerned over the fact that the implant dissolves and expressed fear over where the contents would go and potential health effects related to biodegradation or increased drug exposure, including potential lasting effects on fertility. Participants expressed negative views of the biodegradable nature of the implant in 30 of 50 FGDs with women and 6 of 10 FGDs with men across the two countries.

*Of course*, *its disappearance will cause problems*. *Because if it disappears in the body*, *it means it will stay forever because it will mix with the blood*.*–*Non-user in Central Region, Burkina Faso

Similarly, many providers mentioned that the dissolving of the implant could cause confusion and fear among family planning clients. A few providers mentioned that women already fear that contraceptive implants can “disappear” in the body, so a method that does dissolve could reinforce this myth. Several providers in Uganda specifically mentioned women’s fear that the implant would travel to the heart.

*I think it would not work*. *Because now days even with the existing one*, *mothers have a myth that it can move and disappear into the heart*. *So if you bring this one that gets absorbed mothers will not like it in fact*. *They will say it is in their hearts*. *Even people’s hearts will start paining*. *–*Nurse in Central Region, Uganda

In several FGDs with women and men in Burkina Faso, participants worried about women, especially those who are uneducated, forgetting to renew their method due to a combination of the odd 1.5 year-duration and of the fact that removal would not be necessary. As with the other new hormonal methods, irregular bleeding was also mentioned as an unappealing feature by a number of participants.

#### Advantages and disadvantages of the longer-acting injectable

In 37 of 50 FGDs with women, 7 of 10 FGDs with men, 30 of 37 provider IDIs, and 8 of 15 key informant interviews, participants found the six-month duration of the longer-acting injectable to be an advantage. Several women and many providers noted the increased convenience associated with the reduced number of clinic visits required. A number of participants, mostly women and providers, also commented on liking the injectable delivery system because it is familiar, discreet, and minimally invasive. A non-using mother of six in Boucle du Mouhoun Region, Burkina Faso noted:

*I think that the injectable here will be popular, there are many more women who use the injectable than the other methods here…the injectable here, they like that much more than the other (methods)*.

One common drawback of the injectable identified by respondents was the potential for side effects, especially bleeding irregularities. Women, providers, and some men were well aware of the bleeding side effects associated with the existing three-month product and did not like the prospect of these with a new injectable. Many women and providers and several men were particularly concerned about the possibility of experiencing such side effects for an extended period because they saw no way to reverse them since the method cannot be removed. A few mentioned the need for an “antidote”. One Ugandan man said:

*I may take my wife for it and she reacts badly to it, she will still have to take all the six months in that condition to maybe even die because there is no way they can reverse it. If only there was a way of reversing the condition it would be fine but since it is not there, it is a bad method*.

- 27-year-old father of one whose wife uses pills in Central Region, Uganda

Several providers and key informants were also concerned about delayed return to fertility with a longer acting injectable since this is already a problem with the three-month product. A gynecologist in East Central Region, Uganda said:

*The challenge would be to fix the time for return to fertility. The current injectable is 10 months. For this new injectable, how long will it take? Users need facts about return to fertility. What if it takes 2 years? People should know the exact period*.

#### Advantages and disadvantages of non-surgical permanent contraception

Women and providers noted that the non-surgical nature of the procedure was an advantage, especially in Uganda. Some mentioned that it would be less painful than surgery and would not leave a scar. Others mentioned that it eliminates surgical risk and would be more appealing to women who are afraid of surgery. A few providers in both countries said that more service delivery points may be able to administer a non-surgical option, which could increase access to permanent contraception. Several women and a few men commented positively on the fact that this method would not affect menstruation and, in Uganda, some women and providers said it was good that the method would not contain hormones.

Reactions to the idea of a permanent contraceptive were mixed. Overall, participants across groups welcomed the idea of a permanent option for couples who do not want more children, but many expressed some level of discomfort with an option that would not be reversible. A medical officer in Burkina Faso noted:

*Permanent methods, as of now they have not become the norm in our clients’ minds. It can be offered like other methods, but as to acceptability and uptake, that is a whole other ball game*.

A few participants in Uganda were concerned about potential health effects of the procedure including implications of blocking the fallopian tubes. Several Ugandan providers mentioned that the procedure would require a skilled provider and that it could easily be performed incorrectly.

*There are chances that service providers may not do the right thing*. *Remember you are out and just using a gadget to direct the foam in so if you have not targeted the tubes the foam may not be there and the woman may conceive*.–Provider in East Central Region, Uganda

## Discussion

The purpose of this research was to solicit end user input to inform and guide ongoing contraceptive product development efforts. Overall, the study results provide strong support for continued investment in the development of longer-acting methods. A large majority of current contraceptive users and non-users in both Uganda and Burkina Faso expressed interest in using one of the new methods. As expected, we did not find universal acceptability or lack of acceptability for any of the methods included in the study. Multivariable analysis showed the strongest and most consistent predictors of interest across the two countries in new long-acting methods were desire for a method that lasts longer than one year and acceptability of contraceptive-induced amenorrhea. It should be noted that this research did not include new short-acting or peri-coital methods, so the former association is not unexpected. Respondents identified clear advantages and disadvantages for each of the methods being considered. Some trends in user preferences did emerge that should be taken into consideration as future development decisions are taken.

Women expressed greatest interest in using methods similar to those they are familiar with–the longer-acting injectable and the single-rod implant. Though not necessarily surprising, this result is still informative as use of, or familiarity with, a method doesn’t necessarily equate to satisfaction with it or ensure continued use in the context of a different method mix. Women particularly liked the extended duration of these new options; however, they also expressed concerns over potential side effects. Of particular concern is the potential for irregular menstrual bleeding, which is one of the main reasons for early discontinuation of existing injectables and implants [22]. While the longer duration of the new methods may reduce the need for resupply and perhaps reduce costs associated with method use, product development efforts should prioritize research into reducing menstrual side effects associated with progestin-only methods.

Compared with new injectables and the non-biodegradable implant, women indicated less interest in using the new IUDs included in this study. While 44 and 78% of women in Burkina Faso and Uganda are aware of IUDs, less than 1% of women currently use them in either setting [17,18]. Concerns about the new IUDs included in the study stemmed from negative perceptions of the existing copper IUD, the potential for increased menstrual bleeding, and discomfort with the idea of uterine placement. Slightly more women in both countries said they would be interested in using the LNG-IUS compared with the new copper IUD, and some women liked that the LNG-IUS could be used to treat dysmenorrhea. Respondents’–both women’s and providers’–conflicting response to the LNG-IUS’s potential to cause amenorrhea again highlights importance of changes in menstrual bleeding on method acceptability and the potential impact differences in preferences may have on uptake. The potential for early removal was also important to users, however, comments from both women and providers demonstrate that the products’ labeled duration of effectiveness is an important factor in how they view the method. An IUD lasting 10 years was considered, “too long” for some respondents indicating that the possibility of early removal was either not understood or not practically achievable in their view. While concerns about product duration were raised most frequently in the context of the 10-year copper IUD, the effect of labeled duration on user and provider perceptions of all long acting methods is important to consider.

The two most novel new methods included in the study–the biodegradable implant and non-surgical permanent contraception–elicited strong reactions from many respondents. Concerns expressed during the qualitative phase about the biodegradable nature of the biodegradable implant were reflected in the relatively low proportions of survey respondents expressing interest in using the method. Many women wondered where the implant would “go” when it biodegrades and whether the dissolving would be a health concern. Providers pointed out that these concerns may lead to damaging myths about the product. Reactions to the idea of NSPC were mixed in both countries, however, more respondents in Uganda viewed the possibility of such a method positively. In the qualitative discussions, women and providers felt the method would overcome important barriers to the existing permanent option, namely fear of surgery and provision of surgical contraception.

An important limitation to this early “acceptability” research is that it involves asking respondents to evaluate potential products based on limited information and without actually using them. A related weakness is poor predictive validity, which is compounded by the potential for social desirability bias and the fact that eventual demand and use will also be influenced by other factors such as demand-generation efforts, quality counseling, or geographic and economic access. Further research is needed to assess these important factors.

Even with these limitations, however, we do believe that asking potential users about their preferences and hypothetical interest in using new methods under development has value. This type of contraceptive “market research” has largely been absent in prior contraceptive development efforts resulting in current methods that don’t meet need the contraceptive needs of all women and men [23,24]. Lessons learned from the contraceptive field have been used by developers of multipurpose preventive technologies (MPTs), however, to include acceptability research as part of the product development process [25–27]. At a minimum our results are directional. We see a substantially stronger interest in injectables and implants compared with IUDs. We also uncovered a strong interest in a non-surgical option of permanent contraception among a certain population of users. Being able to identify the most receptive acceptors will be crucial for the success of the methods. We also collected important insights into potential opportunities and barriers for the introduction of the new products once they reach the market. For example, our results highlight the need for carefully crafted marketing and counseling messages that will alleviate potential fears about the biodegradable nature of the biodegradable implant. Similarly, we found that some women are not only accepting but welcoming of the potential for the LNG-IUS to induce amenorrhea and alleviate problematic menstruation. Framing this non-contraceptive effect as a benefit may be important for market positioning. As with any product innovation, messaging should be carefully tailored to prevent misinformation and to help clients weigh in advantages and disadvantages. Based on our findings, new contraceptive products will be highly acceptable if important improvements are made and misconceptions can be addressed.

Developing new contraceptive technologies requires substantial time, dedication, and investment. Ensuring that the products that make it through the long development pipeline are ultimately liked and used by women and couples requires that their needs and preferences are considered early in the process.

## Supporting information

S1 Appendix Method descriptions and images used in the PMA2020 Round 4 surveys in Burkina Faso and Uganda. (PDF)

S2 Appendix Method descriptions used in the focus group discussions (FGDs) with women and men and in-depth interviews (IDIs) with family planning providers. (PDF)
