# Correction: Potential user interest in new long-acting contraceptives: Results from a mixed methods study in Burkina Faso and Uganda

**DOI:** 10.1371/journal.pone.0223090

**Published:** 2019-09-23

**Authors:** The PLOS ONE Staff

There is an error in affiliation 1 for authors Rebecca L. Callahan and Amelia C. L. Mackenzie. The correct affiliation 1 is: Contraceptive Technology Innovation, FHI 360, Durham, NC, USA.

There is an error in affiliation 2 for author Aurélie Brunie. The correct affiliation 2 is: Health Services Research, FHI 360, Washington DC, USA.

The publisher apologies for the errors.

## References

[1] Callahan RL, Brunie A, Mackenzie ACL, Wayack-Pambè M, Guiella G, Kibira SPS, et al (2019) Potential user interest in new long-acting contraceptives: Results from a mixed methods study in Burkina Faso and Uganda. PLoS ONE 14(5): e0217333 10.1371/journal.pone.0217333 31136612 PMC6538161

