# Supplementary material for: Potential user interest in new long-acting contraceptives: Results from a mixed methods study in Burkina Faso and Uganda
Source: PLoS One. 2019 May 28;14(5):e0217333. doi: 10.1371/journal.pone.0217333 (PMC6538161; doi:10.1371/journal.pone.0217333)
Supplement: S1 Appendix — (PDF) [file pone.0217333.s001.pdf]

Supporting Information Appendix 1. Method descriptions and images used in the PMA2020 Round 4 surveys in Burkina Faso and Uganda

Now I would like to talk with you about methods that are not yet widely available in (Uganda or Burkina Faso) or that are currently being developed. For each method, I will first describe it and then ask if you would be interested in using it at some point in the future. All these methods are highly effective at preventing pregnancy.	
**Longer-lasting injectable:**<br>This method is an injection that a woman would get from a health provider. It would prevent pregnancy for six months.<br>While using this method, a woman may have irregular periods or her period may stop. If a woman has these side-effects, they cannot be stopped until the end of the six months.<br>This method would contain hormones, substances that change how the body functions, like in existing contraceptive methods such as the pill, the injectable, or the implant.	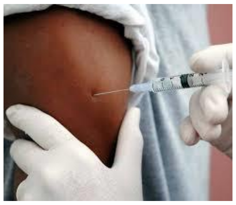
**Longer-lasting single rod implant:**<br>This method is a single rod that would be placed in a woman's arm by a health provider. It would prevent pregnancy for 5 years but could be removed by a health provider at any time.<br>While using this method, a woman may have irregular periods.<br>This method would contain hormones, substances that change how the body functions, like in existing contraceptive methods such as the pill, the injectable, or the implant.	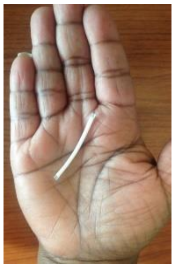
**Dissolving implant:**<br>This method is a single rod that would be placed in a woman's arm by a health provider. It would prevent pregnancy for one and a half years.<br>This method would dissolve over time so it would not need to be removed, however, it could be removed by a health provider during the first year.<br>While using this method, a woman may have irregular periods.<br>This method would contain hormones, substances that change how the body functions, like in existing contraceptive methods such as the pill, the injectable, or the implant.	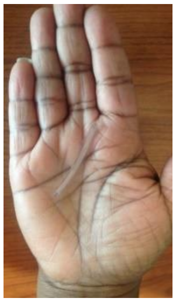
**IUD with hormones:**<br>This is a method where a loop or coil is placed inside a woman's womb by a health provider. It would prevent pregnancy for 5 years but could be removed by a health provider at any time.<br>While using this method, a woman may have a lighter period or her period may stop. This method can also be used as a treatment for heavy and painful menstruation. A woman using this method may experience some pain and discomfort for a short time after it is placed.<br>This method would contain hormones, substances that change how the body functions, like in existing contraceptive methods such as the pill, the injectable, or the implant.	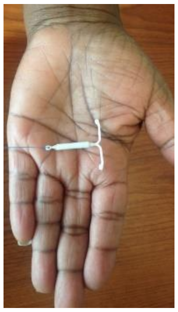

**New IUD without hormones:**<br>This is a method where a loop or coil is placed inside a woman's womb by a health provider. It would prevent pregnancy for 10 or more years but could be removed by a health provider at any time.<br>This method would be different from the current IUD in its shape or size. While using this method, a woman may have heavier periods. A woman using this method may experience some pain and discomfort for a short time after it is placed.<br>This method would not contain hormones.	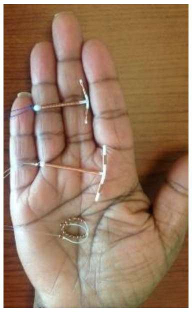
**Permanent method:**<br>A health provider would perform a procedure on the womb that would permanently prevent pregnancy. It would not be an operation.<br>Afterward, the woman may need to return to the health provider for an exam to confirm the method is working completely.<br>This method would not affect a woman's period.<br>This method would not contain hormones.	*No image*

After each method description women were asked, "If this method were available, would you be interested in using it?", to which the answer options were:

Definitely would use it,
Probably would use it,
Probably would not use it,
Definitely would not use it, and
No response.
