# Supplementary material for: Potential user interest in new long-acting contraceptives: Results from a mixed methods study in Burkina Faso and Uganda
Source: PLoS One. 2019 May 28;14(5):e0217333. doi: 10.1371/journal.pone.0217333 (PMC6538161; doi:10.1371/journal.pone.0217333)
Supplement: S2 Appendix — (PDF) [file pone.0217333.s002.pdf]

Supporting Information Appendix 2. Method descriptions used in the focus group discussions (FGDs) with women and men and in-depth interviews (IDIs) with family planning providers

<table>
<tr><td colspan="2">I'd like to get your feedback on some new methods that are being developed. They may become available to women like you in the future, but they are not ready yet. There are six methods. For each method I will first describe it and then ask a few questions about it. All the methods we are going to talk about will be safe and highly effective at preventing pregnancy.</td></tr>
<tr><td>New copper IUD:<ul><li>This method is a device that would be placed inside the womb by a health provider.</li><li>This method would be similar to the copper IUD/LOCAL NAME but it would have a different shape or size.</li><li>It would stop women from becoming pregnant for 10 or more years but you could have it removed at any time.</li><li>While they are using this method, some women may have heavier periods.</li><li>The IUD could come out of the womb, though this would be rare. Some women using this method may experience some pain and discomfort for a short time after it is placed.</li><li>It would not contain any substance that changes how your body functions.</li></ul></td><td>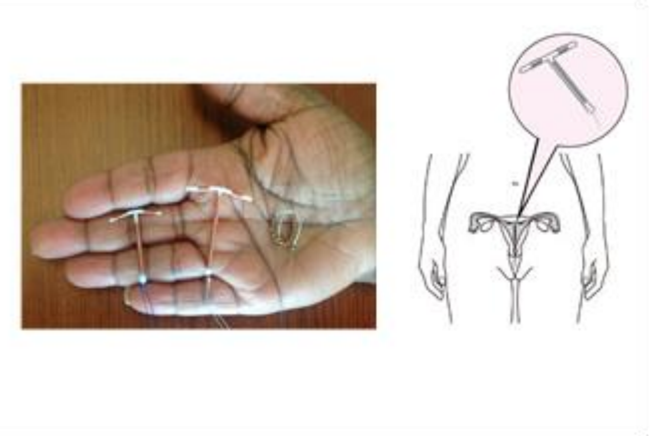</td></tr>
<tr><td>IUD with hormones:<ul><li>This method is a device that would be placed inside the womb by a health provider.</li><li>This method would be similar to the copper IUD/LOCAL NAME but would contain substances that change how your body functions like in existing contraceptive methods such as the pill, the injectable, or the implant.</li><li>It would stop women from becoming pregnant for at least 5 years, but could be removed at any time.</li><li>While they are using this method, some women may bleed less when they have their period or their period may stop.</li><li>This method can also be used as a treatment for heavy and painful menstruation.</li><li>The IUD could come out of the womb, though this would be rare. Some women using this method may experience some pain and discomfort for a short time after it is placed.</li></ul></td><td>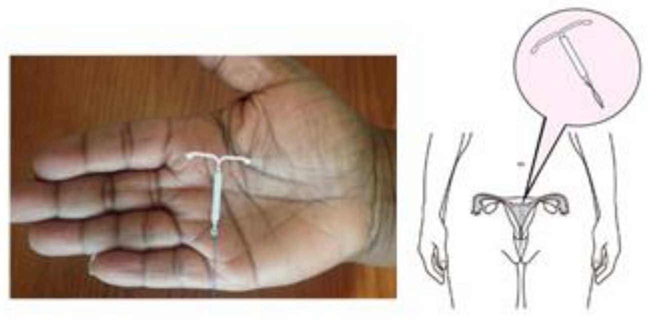</td></tr>
</table>

<table>
<tr>
<td>

**Longer-lasting single rod implant:**

- This method is a rod that would be placed in a woman’s arm by a health provider.
- It would be similar to current types of implants but would protect from pregnancy longer.
- It would stop women from becoming pregnant for at least 5 years, but could be removed at any time.
- While they are using this method, some women may have irregular periods.
- This method would contain substances that change how your body functions like in existing contraceptive methods such as the pill, the injectable, or the implant.

</td>
<td>

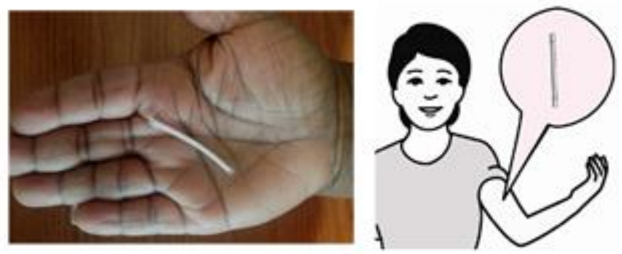

</td>
</tr>
<tr>
<td>

**Biodegradable implant:**

- This method is a rod that would be placed in a women’s arm by a health provider.
- It would stop women from becoming pregnant for one and a half years.
- While they are using this method, some women may have irregular periods.
- This method would dissolve over time so it would not need to be removed, however it could be removed by a health provider during the first year.
- This method contains substances that change how your body functions like in existing contraceptive methods such as the pill, the injectable, or the implant.

</td>
<td>

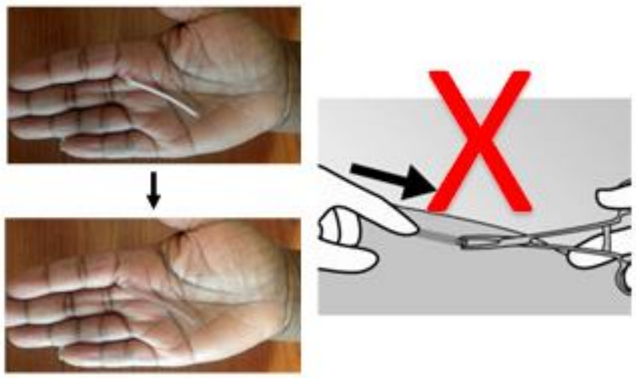

</td>
</tr>
</table>

<table>
<tr>
<td>
Longer-lasting injectable:
<ul>
<li>This method is an injection that women would get from a health provider.</li>
<li>This method would be similar to [INJECTION METHOD(S) CURRENTLY AVAILABLE], but women would only need a new injection after six months instead of three.</li>
<li>It would stop women from becoming pregnant for 6 months.</li>
<li>While they are using this method, some women may have irregular periods or their period may stop. If a woman has these side-effects, they cannot be stopped until the end of the six months.</li>
<li>This method would contain substances that change how your body functions like in existing contraceptive methods such as the pill, the injectable, or the implant.</li>
</ul>
</td>
<td>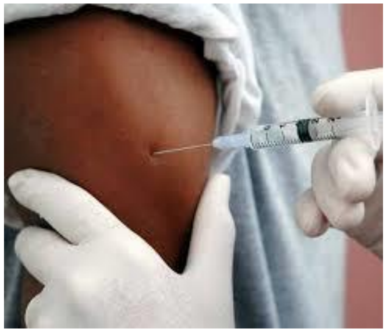</td>
</tr>
<tr>
<td>
Non-surgical permanent contraceptive:
<ul>
<li>This method is a permanent contraceptive for women who do not want any more children.</li>
<li>It would not require surgery but would require a health provider to insert a drug into the uterus to block the tubes.</li>
<li>Afterward, the woman may need to return to the health provider for an exam to confirm the method is working completely.</li>
<li>It would protect from pregnancy forever.</li>
<li>This method should not affect a woman’s period.</li>
<li>This method does not include any substance that changes how your body functions.</li>
</ul>
</td>
<td>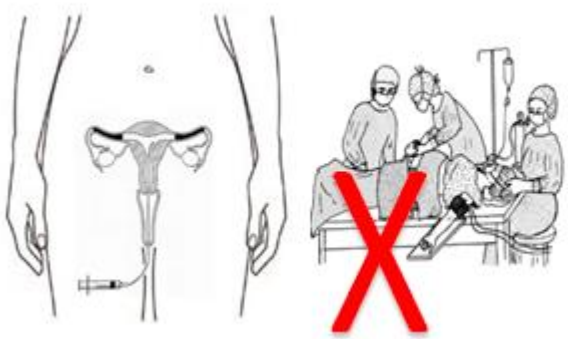</td>
</tr>
</table>
